# Real-time hybrid angular-interrogation surface plasmon resonance sensor in the near-infrared region for wide dynamic range refractive index sensing

**DOI:** 10.1038/s41598-023-42873-w

**Published:** 2023-09-20

**Authors:** Hidenori Koresawa, Kota Seki, Kenji Nishimoto, Eiji Hase, Yu Tokizane, Taka-Aki Yano, Taira Kajisa, Takeo Minamikawa, Takeshi Yasui

**Affiliations:** 1 Graduate School of Advanced Technology and Science, Tokushima University, 2-1 Minami-Josanjima, Tokushima, Tokushima 770-8506 Japan; 2 Graduate School of Science, Technology, and Innovation, Tokushima University, 2-1 Minami-Josanjima, Tokushima, Tokushima 770-8506 Japan; 3 Institute of Post-LED Photonics (pLED), Tokushima University, 2-1 Minami-Josanjima, Tokushima, Tokushima 770-8506 Japan; 4 Graduate School of Interdisciplinary New Science, Toyo University, 2100 Kujirai, Kawagoe, Saitama 350-8585 Japan

**Keywords:** Imaging and sensing, Optical metrology

## Abstract

Herein, we integrated angle-scanning surface plasmon resonance (SPR) and angle-fixed SPR as a hybrid angular-interrogation SPR to enhance the sensing performance. Galvanometer-mirror-based beam angle scanning achieves a 100-Hz acquisition rate of both the angular SPR reflectance spectrum and the angle-fixed SPR reflectance, whereas the use of near-infrared light enhances the refractive index (RI) sensitivity, range, and precision compared with visible light. Simultaneous measurement of the angular SPR reflectance spectrum and angle-fixed SPR reflectance boosts the RI change range, RI resolution, and RI accuracy to 10^–1^–10^–6^ RIU, 2.24 × 10^−6^ RIU, and 5.22 × 10^−6^ RIU, respectively. The proposed hybrid SPR is a powerful tool for wide-dynamic-range RI sensing with various applications.

## Introduction

Surface plasmon resonance (SPR)^1,2^ is a resonance absorption phenomenon caused by the coupling of an evanescent wave and collective vibrations of free electrons when light is incident on a glass prism coated with the metal thin film around the incident angle of total internal reflection (namely, Kretschmann configuration). Because SPR depends on the refractive index (RI) near the sensor surface of the film, it functions as a simple, sensitive RI sensor as well as chemical sensor^3,4^. Furthermore, if the sensor surface is modified with an antibody molecular recognition layer, it functions as a biosensor for a target antigen via antibody-to-antigen interaction because such interactions change the effective RI near the sensor surface^5–7^. To measure RI changes occurring in the field of an electromagnetic wave supported by the optical structure of the sensor, SPR sensor includes the resonant mirror sensor^8,9^, the grating coupler sensor^10–12^, the integrated optical Mach–Zehnder interferometer^13,14^, the integrated Young interferometer^15,16^, and the white light interferometer^8,17^. Furthermore, SPR sensors based on novel materials and/or structures have been investigated: gold film^18,19^, gold particle^20,21^, graphene oxide^22^, and nanostructure^23^. In these wide variety of SPR sensors, RI sensitivity and RI resolution are important performance parameters. The RI sensitivity is defined as a change in SPR signal per unit RI whereas the RI resolution refers to the smallest detectable change or difference in the RI that can be accurately measured by SPR.

Because SPR depends on the angle and wavelength of the incident light, SPR sensors are classified into two types: angular-interrogation SPR^3,4,24–26^ and wavelength-interrogation SPR^27,28^. The former acquires the angular spectrum of the SPR dip by measuring the reflectance with respect to the incident angle using monochromatic light. The latter acquires the wavelength spectrum of the SPR dip by measuring the reflectance spectrum using a multichannel spectrometer when broadband light is used for incident light. Although these SPRs have advantages and disadvantages, angular-interrogation SPR is more widely used than wavelength-interrogation SPR owing to its simple and cost-effective setup.

Angular-interrogation SPR is further classified into three types: prism-angle-scanning^4,24,25^, prism-angle-fixed^7^, and multi-channel angle^3,26^. In the prism-angle-scanning type, the angular SPR reflectance spectrum is measured by mechanical angle scanning of the prism; however, acquiring the angular SPR reflectance spectrum in real time is difficult owing to mechanical scanning. In the prism-angle-fixed type, the SPR reflectance is measured in real time at a fixed incident angle because the RI-dependent shift of the angular SPR reflectance spectrum is converted into an RI-dependent change in SPR reflectance at an incident angle close to the linear slope of the SPR dip. However, SPR sensing without acquiring the angular SPR reflectance spectrum often causes a temperature drift of sensor signal in biosensing application^7^. In the multi-channel angle type, a line-focused beam of monochromatic light is incident onto the prism as a collimated beam with various incident angles, and a line image of the reflected light from the prism is subsequently acquired using a CCD camera. The angular SPR reflectance spectrum is extracted from the obtained image without mechanical scanning of the prism; however, the use of the CCD camera limits the dynamic range of light intensity, and its uneven sensitivity among camera pixels leads to additional background noise in the angular SPR reflectance spectrum^29^. Recently, we reported another angle-scanning type of angular-interrogation SPR based on beam scanning of incident light with a galvanometer mirror (GM) and relay lenses, namely beam-angle-scanning type^29^. GM-based beam angle scanning overcomes slow data acquisition inherent in the prism-angle-scanning type, whereas the use of a photodetector improves the dynamic range of light intensity and eliminates the pixel-to-pixel sensitivity unevenness inherent in the multi-channel angle type.

To further boost the total performance of the RI sensing, herein, we newly focus on two interesting aspects of the beam-angle scanning type. The first aspect is real-time dual measurement of the angular SPR reflectance spectrum and angle-fixed SPR reflectance. If the reflectance at a certain beam angle is extracted from a series of angular SPR reflectance spectra continuously acquired by fine, rapid beam scanning, we can acquire a reflectance signal equivalent to that of the angle-fixed type, too. Simultaneous acquisition of the angular SPR reflectance spectrum and angle-fixed SPR reflectance may combine a wide RI range of the prism-angle-scanning type and high RI sensitivity of the prism-angle-fixed type, along with the real-time measurement similar to the multi-channel angle type. In other words, the beam-angle scanning type has a potential to eliminate the problems of the previous three types and enables the integration of their strengths. The second aspect is use of near-infrared (NIR) light instead of visible light for incident light. This is because the angular SPR dip sharpens in the NIR region more than in the visible region^27^. Unfortunately, NIR light is not so compatible with multi-channel angle type because of high cost and poor performance of NIR cameras (for example, InGaAs CCDs) compared with visible camera (for example, Si CCDs); conversely, the performance of NIR photodetectors (for example, InGaAs photodiodes) is comparable to that of visible photodetectors (for example, Si photodiodes). Hence, angular-interrogation SPRs except the multi-channel angle type can benefit from the use of NIR light. In the measurement of the angle-fixed SPR reflectance, the sharpening of the angular SPR dip increases the steepness of the SPR dip slope and enhances the RI sensitivity; however, it exhibits the drawback of a reduced measurable range of RI owing to a steep slope. Conversely, in the measurement of the angular SPR reflectance spectrum, the sharped angular SPR dip expands the measurable range of RI sensing. They are complementary to each other.

In this article, to investigate the effectiveness of two aspects above, we construct the beam-angle-scanning type, angular-interrogation SPR using NIR light for real-time simultaneous acquisition of angular SPR reflectance spectrum and angle-fixed SPR reflectance (we call it real-time hybrid angular-interrogation SPR). In wide-range RI sensing and high-resolution RI sensing of solution samples, we compare the RI sensing performance between measurements of angular SPR reflectance spectrum and angle-fixed SPR reflectance. We also discuss the enhancement of RI sensing performance in the beam-angle-scanning SPR by use of NIR light instead of visible light.

## Principle of operation

**Figure 1:**
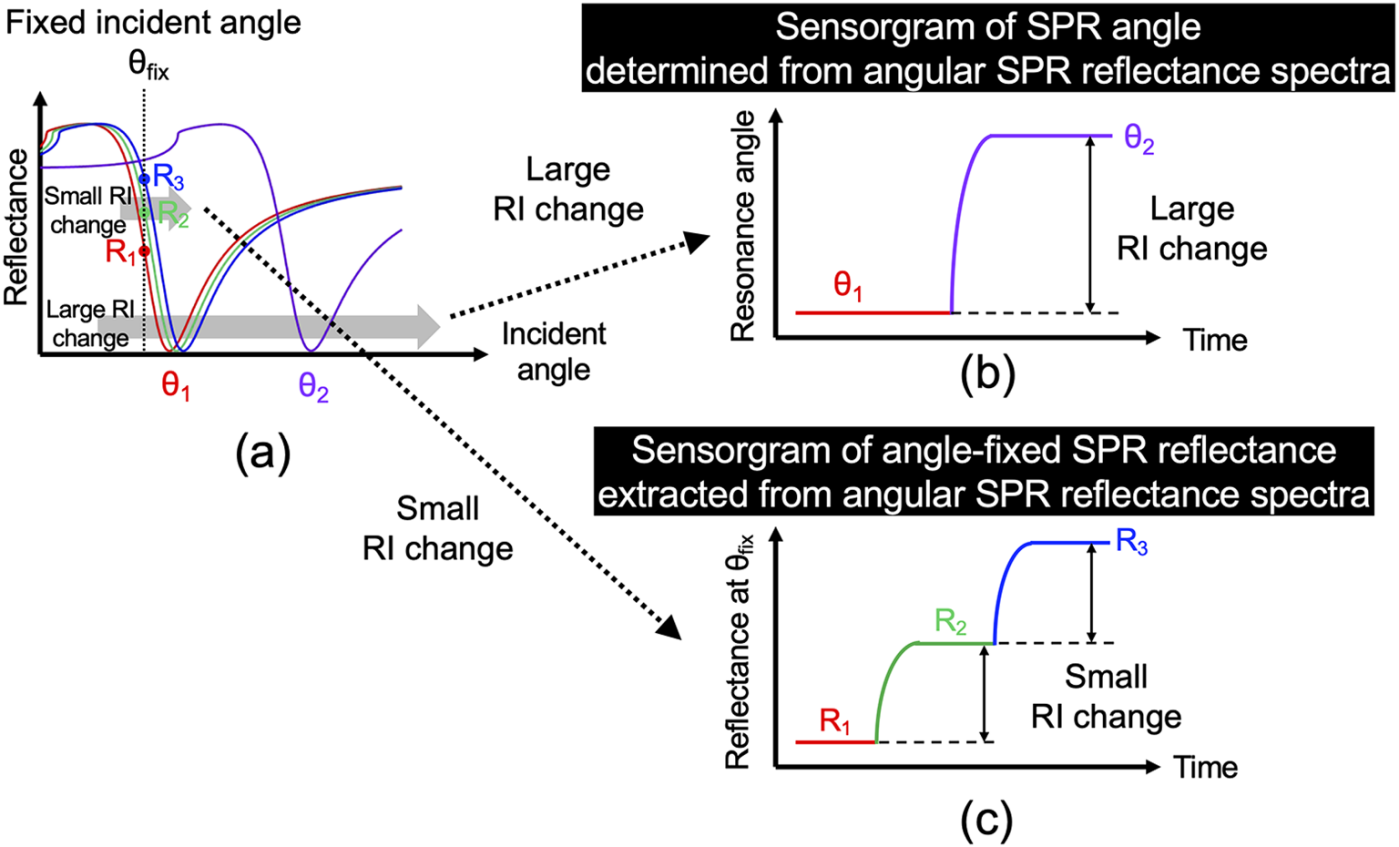
Principle of operation for dual measurement of angular SPR reflectance spectrum and angle-fixed SPR reflectance. (**a**) Dependence of angular SPR reflectance spectrum on large change of sample RI as well as dependence of angle-fixed SPR reflectance on small change of sample RI. (**b**) Sensorgram of SPR angle when the sample RI change is large. (**c**) Sensorgram of the angle-fixed SPR reflectance when the sample RI change is small.

We describe the principle of operation for the real-time dual measurement of the angular SPR reflectance spectrum and angle-fixed SPR reflectance in the beam-angle-scanning type. Figure 1a shows the spectral behavior of the angular SPR dip with respect to the change in the sample RI. The large change in the sample RI is correctly determined from the spectral shift of the angular SPR dip (i.e., *θ*_*1*_ to *θ*_*2*_) because the spectral shift is larger than the spectral width of the angular SPR dip. However, in the case of a small RI change, because the SPR spectral shift is smaller than the SPR spectral width, precisely determining the sample RI from the spectral shift of the angular SPR dip is difficult (i.e., around *θ*_*1*_). Therefore, the measurement of the angular SPR spectrum by the beam-angle scanning is suitable for wide-range RI measurements but not necessarily for high-resolution RI measurements (see Fig. 1b). Conversely, the measurement of angle-fixed SPR reflectance is suitable for high-resolution RI measurements because a small change in sample RI is enhanced by the steep linear slope of the SPR dip, and the resulting change in reflectance can be precisely determined from a change of optical intensity at a fixed incident angle by a photodetector (i.e., *R*_*1*_, *R*_*2*_, to *R*_*3*_). However, when the fixed incident angle is out of the steep linear slope of the SPR dip owing to the large change in sample RI, the measurement of angle-fixed SPR reflectance cannot be used for RI sensing. Therefore, although the measurement of angle-fixed SPR reflectance is suitable for high-resolution RI measurements, it is unsuitable for wide-range RI measurements (see Fig. 1c). Thus, the angle-scanning measurement of the angular SPR reflectance spectrum is complementary to the angle-fixed measurement of the SPR reflectance. The beam-angle scanning type combines the advantages of these two measurements together with real-time measurement.

**Figure 2:**
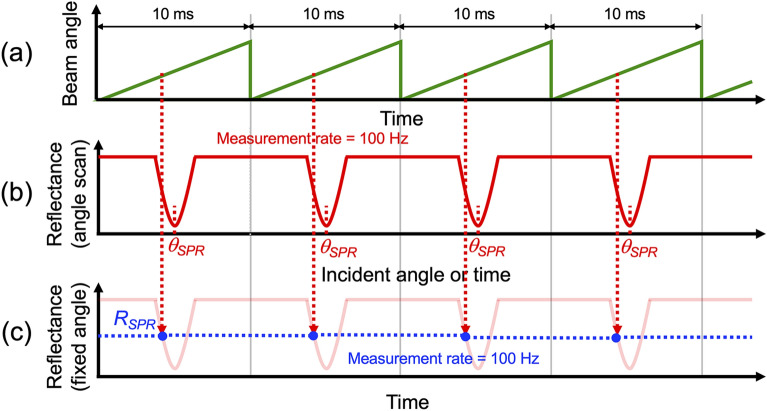
Measurement timing chart with respect to the angle-scanning angular SPR reflectance spectrum and the angle-fixed SPR reflectance. Temporal change of (**a**) beam angle, (**b**) reflectance spectrum, and (**c**) reflectance at the fixed incident angle.

Figure 2 shows a measurement timing chart with respect to the angle-scanning angular SPR reflectance spectrum and the angle-fixed SPR reflectance. The angular SPR reflectance spectrum was acquired in synchronization with the beam angle scanning because it corresponds to a temporal change in reflectance with respect to beam angle scanning (see Fig. 2a and b). The acquisition rate of the angular SPR reflectance spectrum increases up to the beam scanning rate (typically, a few hundred hertz) using GM and relay lenses. Furthermore, the use of GM and relay lenses enables fine beam angle scanning in addition to rapid beam angle scanning. In this case, the high reproducibility of beam angle scanning was secured in repetitive acquisitions of the angular SPR reflectance spectrum. If only the reflectance at a certain beam angle near the SPR dip slope is repetitively extracted from the angular SPR reflectance spectrum, the resulting reflectance becomes equivalent to the prism-angle-fixed type SPR sensor signal (see Fig. 2a and c). The acquisition rate of the angle-fixed SPR reflectance was also equal to the beam scanning rate with the GM. Although the prism-angle-scanning type can acquire both the angular SPR reflectance spectrum and angle-fixed SPR reflectance in a similar manner, its acquisition rate is considerably lower than that of the beam-angle-scanning type due to the mechanical scanning of prism.

## Experimental setup

**Figure 3:**
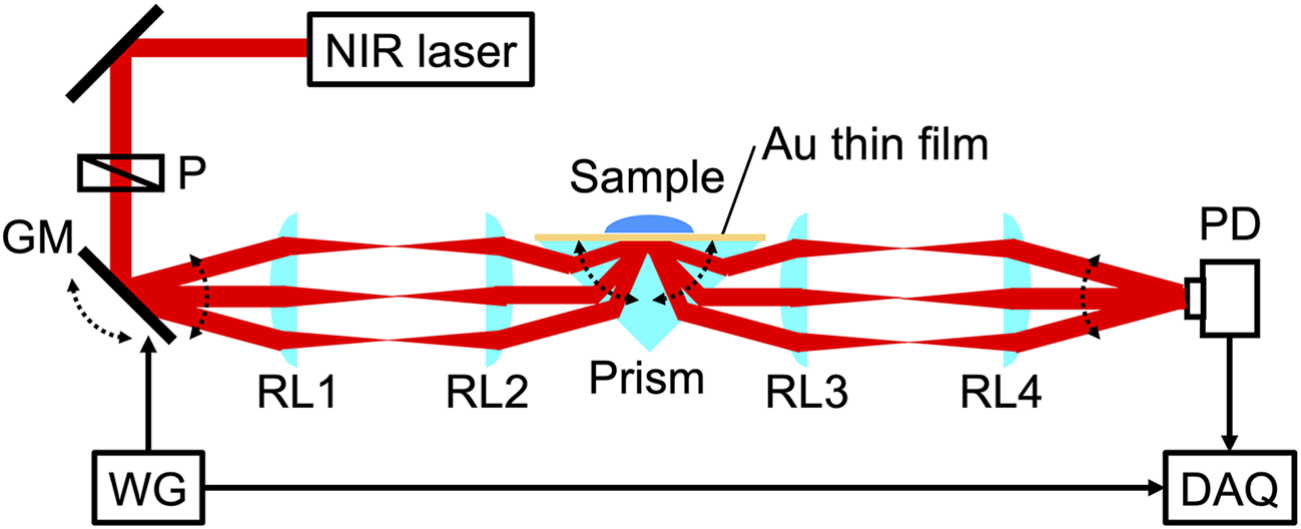
Experimental setup. P, Glan–Taylor polarizer; GM, single-axis galvanometer mirror; RL1, RL2, RL3, and RL4, relay lenses; PD, photodetector; DAQ, data acquisition board; WG, waveform generator.

Figure 3 shows a schematic of the experimental setup for hybrid angular-interrogation SPR. We used a single-frequency distributed-feedback (DFB) fiber laser (Koheras BasiK E15, NKT PHOTONICS, Denmark, wavelength = 1550.12 nm, optical power = 40 mW, polarization = linear) for a monochromatic NIR light source. After passing through a Glan–Taylor polarizer (P, GT10-C, Thorlabs Inc., wavelength = 1050–1700 nm, clear aperture = 10 mm, extinction ratio = 100,000:1), the collimated laser beam was angle-scanned within an optical scan angle range of ± 1.1° at 100 Hz using a single-axis galvanometer mirror (GM, GVSM001-JP/M, Thorlabs Inc., maximum optical scan angle range =  ± 20°, resolution = 0.0008°, bandwidth of triangular wave = 175 Hz) and subsequently passed through a pair of relay lenses (RL1, RL2, focal length = 100 mm, diameter = 40 mm). We used the Kretschmann configuration of a right-angle prism for the RI sensing of a liquid sample. A gold (Au) thin film (thickness = 30 nm) was made on the rear surface of a glass right-angle prism (RPB-30-2L, Thorlabs Inc., glass material = BK7, length = 30 mm, uncoated) using a chromium (Cr) thin film (thickness < 5 nm) for the adhesive layer. The laser beam refracted at the incident surface of the prism and moved toward the prism/Cr/Au interface. The combination of GM and relay lenses enables rapid incident-angle scanning of the collimated laser beam (angle range = 61.8°–64.0°) near the total reflection angle at a fixed position of the prism/gold interface while maintaining beam collimation (beam diameter = 3.6 mm). The angle-scanning reflection beam was relayed using another pair of relay lenses (RL3, RL4, focal length = 100 mm, diameter = 40 mm). The resulting beam was detected by a photodetector (PD, PDA20C2, Thorlabs Inc., wavelength = 800–1700 nm, bandwidth = DC ∼5 MHz, active area = 3.14 mm by 3.6 mm). The output voltage signal from the photodetector and the driving voltage signal for the GM from a waveform generator (WG) were synchronously acquired using a data acquisition board (DAQ, USB-6361, National Instruments Corp., ADC resolution = 16 bit, maximum sample rate = 2 MSample/s). The resolution and sampling interval of the beam incident angle on the prism/Cr/Au interface were set to 0.0005° and 0.0022°, respectively. The angular SPR reflectance spectrum was obtained at 100 Hz from the signals acquired by the photodetector and GM. Simultaneously, the SPR reflectance was extracted at a fixed incident angle on the angular SPR reflectance spectrum each time the angular SPR spectrum was acquired.

## Results

### Wide-range RI sensing

For wide-range RI sensing, we used mixtures of ethanol and pure water with different mixture ratios as a sample for RI sensing. The RI of the sample was calculated from the volume ratio of water (RI = 1.3317 refractive index unit or RIU at 1550 nm) and ethanol (RI = 1.3604 RIU at 1550 nm)^30,31^. We prepared five types of samples with different mixture ratios: 0 EtOH% (pure water; RI = 1.3180 RIU), 2.5 EtOH% (RI = 1.3188 RIU), 5 EtOH% (RI = 1.3196 RIU), 7.5 EtOH% (RI = 1.3205 RIU), and 10 EtOH% (RI = 1.3213 RIU).

**Figure 4:**
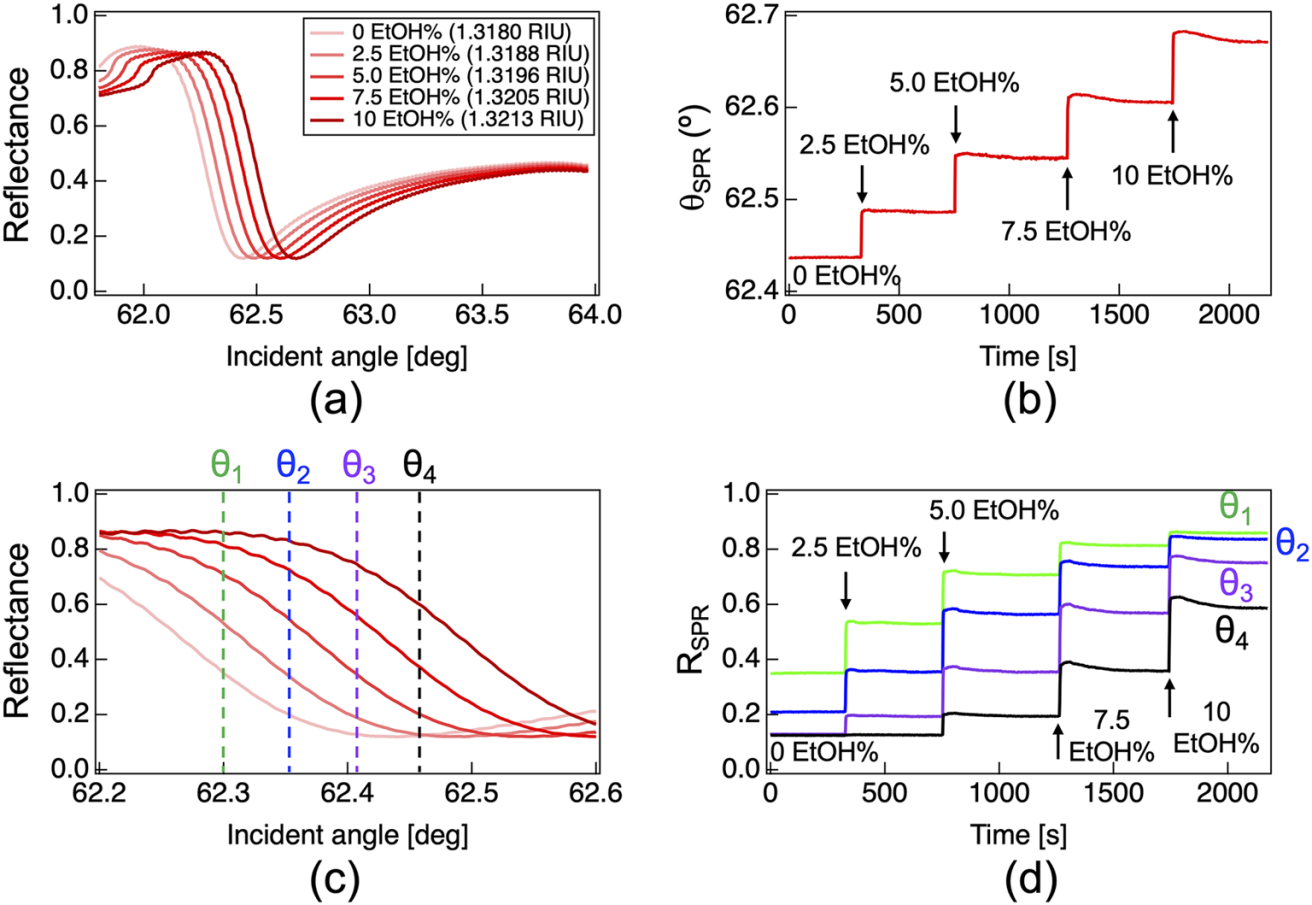
Experimental results of wide-range RI sensing. Sample concentration increased stepwise from 0 EtOH% to 10 EtOH%. (**a**) Angular SPR reflectance spectra and (**b**) its corresponding sensorgram of the SPR reflectance angle (*θ*_*SPR*_). (**c**) Comparison of angular SPR reflectance spectra magnifying the incident angle range of 62.2–62.6° of (**a**) and (**d**) its corresponding sensorgram of the angle-fixed SPR reflectance (*R*_*SPR*_).

First, we measured the angular SPR reflectance spectra of the water/ethanol samples. Figure 4a shows the angular SPR reflectance spectra with respect to different ethanol concentrations (incident angle range = 61.8°–64.0°). The angular SPR dip appears at approximately 62.5°, indicating its dependence on the ethanol concentration. To determine the center angle *θ*_*SPR*_ of the angular SPR dip, curve fitting analysis with a sixth order polynomial function was adopted for the angular SPR reflectance spectra. Figure 4b shows the corresponding sensorgram of *θ*_*SPR*_ when the sample concentration increased stepwise from 0 EtOH% to 10 EtOH%. The step-like behavior with a constant shift in *θ*_*SPR*_ indicates that the measurement of angular SPR reflectance spectrum secures the linearity of the sensor signal in such wide-range RI sensing.

Next, we measured the angle-fixed SPR reflectance *R*_*SPR*_ of the same water/ethanol samples. To select a fixed incident angle, we magnified the angle range of the left slope of the SPR dip in Fig. 4a. Figure 4c shows a comparison of the magnified angular SPR spectra within the incident angle range of 62.2°–62.6°. We extracted the SPR reflectance at four different incident angles from the angular SPR reflectance spectra: *θ*_*1*_ = 62.300°, *θ*_*2*_ = 62.348°,* θ*_*3*_ = 62.405°, and *θ*_*4*_ = 62.462°. In other words, we extracted the reflectance along the green dashed line for *θ*_*1*_, blue line for *θ*_*2*_, purple line for *θ*_*3*_, and black line for *θ*_*4*_ in Fig. 4c. Figure 4d shows the corresponding sensorgram of the reflectance at *θ*_*1*_, *θ*_*2*_, *θ*_*3*_, and *θ*_*4*_ when the sample concentration increased from 0 EtOH% to 10 EtOH%. Although the step-like behavior was confirmed depending on the ethanol concentration, the reflectance shift caused by the sample concentration change was not even for every incident angle. In this manner, angle-fixed SPR reflectance measurement does not secure the linearity of the sensor signal in wide-range RI sensing. A comparison of the sensor signal linearity between the angular SPR reflectance spectrum measurement and angle-fixed SPR reflectance measurements highlights the suitability of the former for wide-range RI sensing.

### High-resolution RI sensing

**Figure 5:**
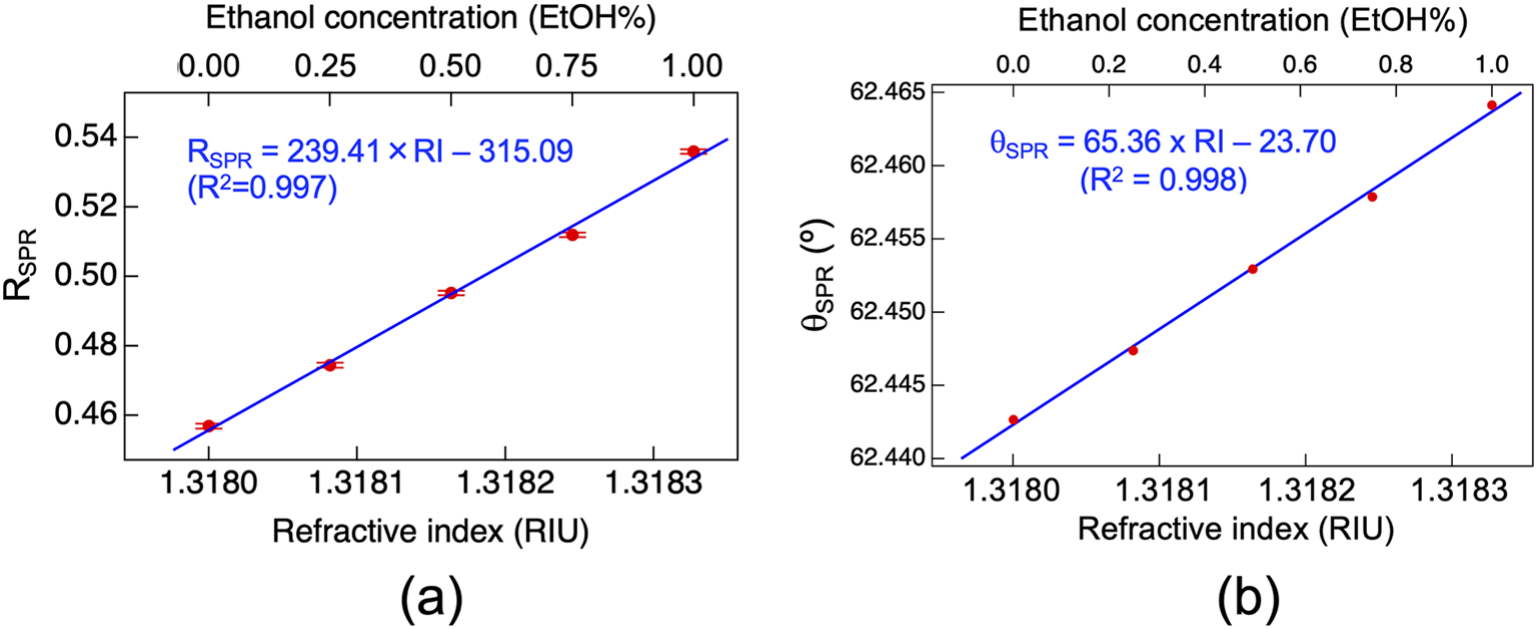
Experimental results of high-resolution RI sensing. Sample concentration increased stepwise from 0.00 EtOH% to 1.00 EtOH%. (**a**) Relation between the sample RI, or ethanol concentration, and the angle-fixed SPR reflectance (*R*_*SPR*_). (**b**) Relation between the sample RI, or ethanol concentration, and the SPR reflectance angle (*θ*_*SPR*_).

For high-resolution RI sensing, we used more dilute water/ethanol samples: the 0 EtOH% (pure water) sample (RI = 1.3180 RIU), the 0.25 EtOH% sample (RI = 1.31808 RIU), the 0.50 EtOH% sample (RI = 1.31816 RIU), the 0.75 EtOH% sample (RI = 1.31825 RIU), and the 1.0 EtOH% sample (RI = 1.31833 RIU). We measured the angle-fixed SPR reflectance *R*_*SPR*_ of the samples. Figure 5a shows the relation between the sample RI or sample concentration and *R*_*SPR*_ when the incident angle was fixed at 62.274°. A linear relationship was observed between the two variables. From the curve fitting analysis, as shown by the blue line in Fig. 5a, the linear relationship was determined to be *R*_*SPR*_ = 239.41RI–315.09, with a coefficient of determination (R^2^) of 0.997. The RI sensitivity was 239.41 1/RIU from the slope coefficient, whereas the RI resolution was estimated to be 2.81 × 10^−6^ RIU from the RI sensitivity and the mean of the standard deviation of the *R*_*SPR*_ at each RI. When RI accuracy was defined as the root mean square error (RMSE) between the experimental data and the fitting slope, a value of 2.86 × 10^−5^ RIU was achieved.

For comparison, we also measured the angular SPR reflectance spectra of the same water/ethanol samples. Figure 5b shows the relation between sample RI or sample concentration and *θ*_*SPR*_. We confirmed the linear relationship between them, in which *θ*_*SPR*_ = 65.36RI–23.70, with an R^2^ of 0.998. The resulting RI resolution and RI accuracy were determined to be 2.24 × 10^−6^ RIU and 5.22 × 10^−6^ RIU, respectively. A comparison of the RI resolution between the angle-fixed SPR reflectance measurement and the angular SPR reflectance spectrum measurement dose not highlight the superiority of the former for high-resolution RI sensing.

## Discussions

We explored junction points using the RI resolution as a shared evaluation criterion for both angle-fixed SPR reflectance measurement and angular SPR reflectance spectrum measurement in high-resolution RI sensing of more dilute water/ethanol samples. However, the angle-fixed reflected light intensity measurement did not yield the anticipated improvement in RI resolution, and we encountered challenges in identifying the optimal junction points for these two measurements. We first discuss the reason for little difference of RI resolution between them. Basically, since the only difference between two measurements is whether the RI-dependent spectral shift of angular SPR dip is measured by the reflectance on the vertical axis or by the angle on the horizontal axis (see Fig. 1a), both should theoretically have the same RI resolution if the difference in instrumentational resolution and/or the analysis uncertainty are negligible. In the angle-fixed SPR reflectance measurement, RI resolution depends on the detection resolution of reflectance measurement in the linear slope of SPR dip. In this case, it is possible to take advantage of the wide dynamic range of optical intensity measurement in the photodetector although voltage resolution of the following data acquisition board may limit the actual dynamic range. Also, it is sensitive to environmental disturbances such as various noise, laser intensity fluctuation, and temperature drift of sensor signal because such environmental disturbances fluctuate the optical intensity. Especially in the present demonstration, the residual temperature drift of the sensor signal significantly compromises the RI resolution in angle-fixed SPR reflectance measurement. One possible method to suppress the temperature drift is through precise temperature control of the sample, sample cell, and/or sensor surface. Another potential method is to introduce active-dummy temperature compensation based on the balanced detection of SPR-sensitive *p*-polarized light and SPR-insensitive *s*-polarized light. Conversely, in the angular SPR reflectance spectrum measurement, RI resolution depends on the determination uncertainty of *θ*_*SPR*_ by the curve fitting analysis of the measured angular SPR reflectance spectrum. Since the RI-dependent spectral shift of angular SPR dip is relatively small compared with the spectral bandwidth of angular SPR dip, the curve fitting analysis may contain some uncertainty in the determination of extremely small *θ*_*SPR*_ shift. However, it is less sensitive to environmental disturbances benefiting from the curve fitting analysis to the whole reflectance spectrum of angular SPR dip. Based on these considerations, we initially though that the angle-fixed SPR reflectance measurement was more advantageous in terms of high-resolution RI sensing; however, the experimental results suggest that the angle-fixed SPR reflectance measurement and the angular SPR reflectance spectrum measurement were actually similar to each other. One possible reason for the good uncertainty in the determination of *θ*_*SPR*_ shift is due to the fact that the angular SPR reflectance spectrum measurement was performed in the condition of good signal-to-noise ratio (SNR) due to a “bright measurement” using a total reflection. This ideal experimental condition may have improved the uncertainty. The main reason for the failure to identify optimal junction points for these two measurements, as mentioned above, is the influence of temperature drift in the sensor signal. Therefore, we believe that implementing the aforementioned techniques to address this issue will enable us to find the optimal junction points. This aspect will be the primary focus of our future work.

**Figure 6:**
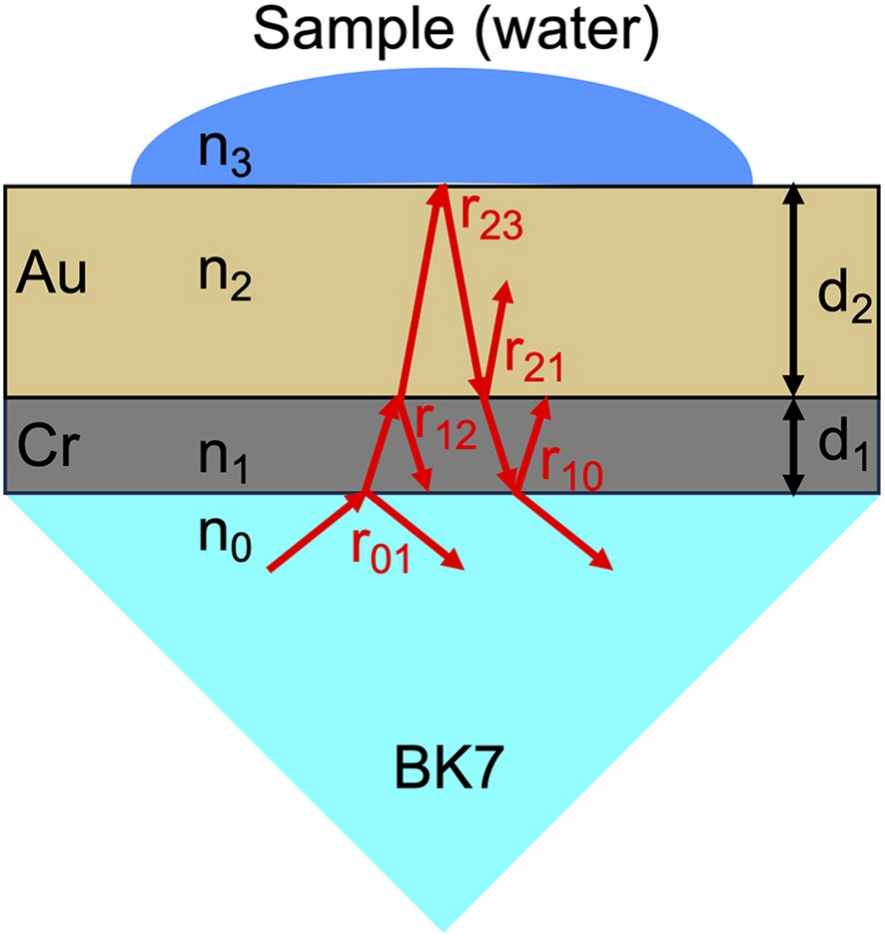
Optical model for numerical calculations based on the Kretschmann configuration for the four-layer model.

We next discuss the comparison of GM-based beam-angle-scanning type between NIR and visible regions. We previously demonstrated a similar GM-based beam-angle-scanning type in the visible-light region for rapid RI sensing and biosensing^29^. Because the previous beam angle scanning is similar to the present one except for the use of monochromatic visible light (wavelength = 633 nm), it has an option for real-time dual measurement of the angular SPR reflectance spectrum and angle-fixed SPR reflectance, which was not mentioned in the previous article. Herein, we discuss the performance of the hybrid angular-interrogation SPR between the visible and NIR regions based on theoretical calculations^7^. The optical model for numerical calculations is based on the Kretschmann configuration for the four-layer model, as shown in Fig. 6. The complex reflectance coefficient *r*_*jk*_ at each interface is expressed by

1 $$\begin{array}{*{20}c} {r_{jk} = \frac{{n_{k} cos\theta_{j} - n_{j} cos\theta_{k} }}{{n_{k} cos\theta_{j} + cos\theta_{k} }},} \\ \end{array}$$

where *n* is the refractive index of the medium, *θ* is incident angle, and the subscripts *j,k* are the medium which light is incident, respectively. The relationship between *θ*_*j*_ and *θ*_*k*_ is given by

2 $$n_{j} sin\theta_{j} = n_{k} sin\theta_{k} .$$

The complex reflectance coefficient *r*_*0123*_ in the four-layer model of BK7/Cr/Au/sample is given by

3 $$r_{0123} = \frac{{r_{01} + r_{123} e^{{ - i \cdot 2\beta_{1} }} }}{{1 + r_{01} r_{123} e^{{ - i \cdot 2\beta_{1} }} }},$$

and the complex reflectance coefficient *r*_*123*_ in the three-layer model of Cr/Au/sample is given by

4 $$r_{123} = \frac{{r_{12} + r_{23} e^{{ - i \cdot 2\beta_{2} }} }}{{1 + r_{12} r_{23} e^{{ - i \cdot 2\beta_{2} }} }},$$

where *β*_*1*_ and *β*_*2*_ are phase delay caused by medium 1 (Cr thin film) and medium 2 (Au thin film), and are given by the following equations

5 $$\beta_{1} = \frac{{2\pi d_{1} n_{1} cos\theta_{1} }}{\lambda },$$

6 $$\beta_{2} = \frac{{2\pi d_{2} n_{2} cos\theta_{2} }}{\lambda },$$

where *d*_*1*_ and *d*_*2*_ are thickness of medium 1 and 2, and λ is wavelength of incident light. The reflectance R for incident light in the optical model is given by

7 $$R = \left| {r_{0123} } \right|^{2} .$$

Here, the RI at 633 nm were set to be 1.515 for the prism (BK7), 3.140 + 3.332i for Cr, 0.183 + 3.433i for Au, and 1.3334 for pure water, respectively, based on literature values^32–34^; we used actual values for thickness of Cr and Au thin films (= 3 nm for Cr and 50 nm for Au). Similarly, the RI at 1550 nm were set to be 1.501 for the prism (BK7), 3.668 + 4.180i for Cr, 0.524 + 10.742i for Au, and 1.318 for pure water, respectively; thickness of Cr and Au thin films on the prism was 3 nm for Cr and 30 nm for Au^29^.

**Figure 7:**
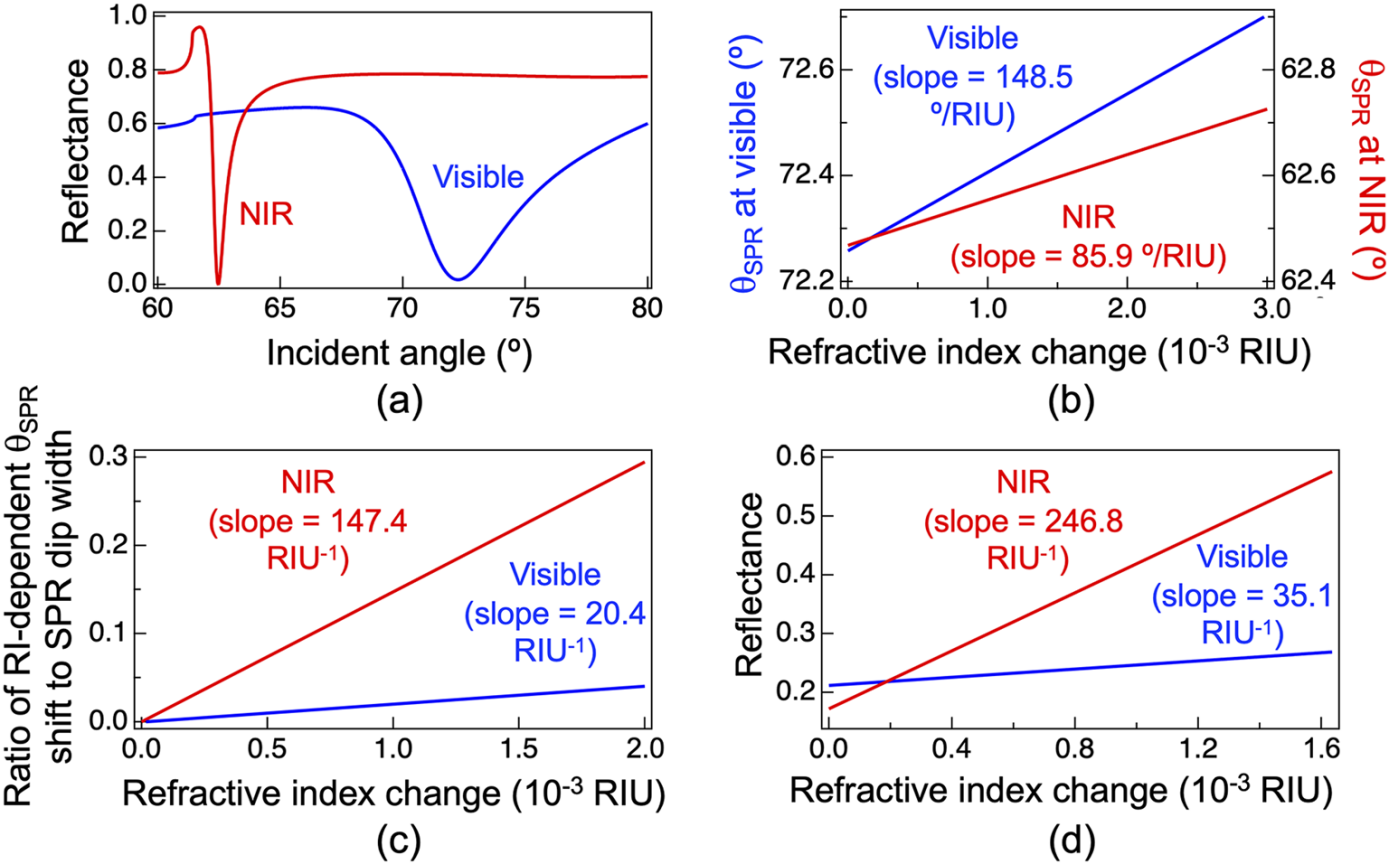
Comparison of sensing performance between the visible hybrid angular-interrogation SPR (wavelength = 633 nm, blue line) and NIR one (wavelength = 1550 nm, red line) based on theoretical calculations for Kretschmann configuration of the four-layer model^7^ when a water is used as a sample. (**a**) Comparison of angular SPR reflectance spectra between visible and NIR regions. (**b**) RI-change-dependence of the angular SPR reflectance angle (*θ*_*SPR*_) at visible and NIR regions. (**c**) Ratio of RI-dependent *θ*_*SPR*_ shift to SPR dip shift with respect to RI change at visible and NIR regions. (**d**) RI-change-dependence of SPR reflectance at 633 and 1550 nm. The sample RI is increased from the pure water (RI = 1.3334 RIU at 633 nm and 1.3317 RIU at 1550 nm).

We first considered the difference in the angular SPR reflectance spectrum measurement between the visible and NIR regions. The blue and red lines in Fig. 7a show the angular SPR reflectance spectra at 633 nm and 1550 nm, respectively, when pure water is used as a sample. The angular SPR dip at 1550 nm was 6 times sharper than that at 633 nm. The RI-dependent shifts of the angular SPR dip *θ*_*SPR*_ at 633 nm and 1550 nm are indicated by the blue and red lines in Fig. 7b. Here, the horizontal coordinate is scaled with respect to the RI change from the pure water. The linear slope at 1550 nm was 1.7 times less steep than that at 633 nm. In other words, such a sharp SPR dip and a less steep slope at 1550 nm are useful for wide-range RI sensing within the limited angle range of GM-based beam scanning. For example, when *θ*_*SPR*_ can be determined within an incident angle range of 65°–75°, the maximum range of RI change is expanded to be 0.116 RIU from the slope of Fig. 7b. In this way, since both SPR dip width and RI-dependent spectral shift depend on wavelength, the figure of merit (FOM) in angular-interrogation SPR is defined as the ratio of the RI-dependent spectral shift to the SPR dip width with respect to sample RI change from the pure water^35^. The blue and red lines in Fig. 7c show the ratio with respect to the sample RI change at 633 nm and 1550 nm, respectively; here, the slope coefficient is corresponding to the FOM value. The FOM value in the NIR region tends to be relatively large (= 147.4/RIU) despite the small RI-dependent spectral shift, due to the narrow width of the SPR dip. Conversely, the FOM value in the visible region tends to be relatively small (= 20.4/RIU) despite the large RI-dependent spectral shift, due to the broad width of the SPR dip. From comparison between them, the FOM value at 1550 nm is seven times larger than that at 633 nm. Although the FOM value in the visible angular-interrogation SPR has been enhanced by use of low-refractive-index porous silica film^35^, its enhanced factor was remained around 2 and the resulting FOM value is still lower than that of NIR angular-interrogation SPR. Therefore, NIR angular-interrogation SPR has the advantage of wide-range and high-precision RI sensing over visible angular-interrogation SPR.

Next, we considered the angle-fixed SPR reflectance measurement. The RI-dependent SPR reflectance at a fixed incident angle also depends on both the SPR dip width and the RI-dependent SPR dip shift. The blue and red lines in Fig. 7d show the RI-dependent reflectance shifts at 633 and 1550 nm, respectively. The RI-dependent SPR reflectance shift at 1550 nm is seven times larger than that at 633 nm. In other words, such a large reflectance shift is effective for high-resolution RI sensing. Interestingly, the enhanced factor (= 7) between visible and NIR regions in the angle-fixed SPR reflectance measurement are the same as that in the angular SPR reflectance spectrum measurement. This fact supports that the angle-fixed SPR reflectance measurement and the angular SPR reflectance spectrum measurement should theoretically have the same RI resolution above.

We further experimentally compare the RI sensing performance based on beam-angle-scanning angular reflectance spectrum measurement between visible^29^ and NIR regions. Table 1 shows a comparison of RI resolution and RI accuracy between them. The enhancement of RI resolution and RI accuracy was confirmed in the beam-angle-scanning angular reflectance spectrum measurement in the NIR region compared with the visible region. Thus, although it changed slightly from the initial claim, the hybrid angular-interrogation SPR in the NIR region benefits from a wide range, high precision, and high resolution compared with the hybrid angular-interrogation SPR in the visible region.

**Table 1:** Experimental comparison of RI sensing performance based on beam-angle-scanning angular reflectance spectrum measurement between visible and NIR regions.

	Visible region^29^ (wavelength = 633 nm)	NIR region (wavelength = 1550 nm)
RI resolution	2.31 × 10^−5^ RIU	2.24 × 10^−6^ RIU
RI accuracy	8.98 × 10^−5^ RIU	5.22 × 10^−6^ RIU

## Conclusion

Simultaneous measurement of the angular SPR reflectance spectrum and angle-fixed SPR reflectance in the NIR region was demonstrated at 100 Hz through rapid, high-precision beam angle scanning based on a combination of GM and relay lenses. Wide-range RI sensing of water/ethanol solutions was performed within an RI range of 1.3180–1.3213 RIU by angular SPR spectrum measurements. Although the range of the incident angle was set between 61.8° and 64.0° by a used optical scan angle range of ± 1.1° in GM, it could be further expanded to by the complete utilization of the maximum optical scan angle range of GM (= ± 20°) and/or modification of the relay-lens configuration while maintaining the same measurement rate, thereby boosting the wide-range RI sensing. Furthermore, the angle-fixed SPR reflectance measurement achieved an RI resolution of 2.81 × 10^−6^ RIU and an RI accuracy of 2.86 × 10^−5^ RIU in the RI sensing of water/ethanol solutions. However, the achieved sensing performance was comparable or inferior to that of angular SPR reflectance spectrum measurements (RI resolution = 2.24 × 10^−6^ RIU, RI accuracy = 5.22 × 10^−6^ RIU). Although the angle-fixed SPR reflectance measurement still has a margin to further increase the data acquisition rate over kHz or MHz, its merit may be small except for this high acquisition rate compared with the beam-angle-scanning angular SPR reflectance spectrum measurement. Furthermore, the superiority of the combination of hybrid angular-interrogation SPR and NIR light was proven by comparison using visible light. The proposed hybrid angular-interrogation SPR is a powerful tool for wide-dynamic-range RI sensing and biosensing.

## Data Availability

Data underlying the results presented in this paper are not publicly available at this time but may be obtained from Takeshi Yasui upon reasonable request.
